# Health Literacy and Income Mediate Racial/Ethnic Asthma Disparities

**DOI:** 10.3928/24748307-20181113-01

**Published:** 2019-01-18

**Authors:** Ryan G. Seibert, Michael R. Winter, Howard J. Cabral, Michael S. Wolf, Laura M. Curtis, Michael K. Paasche-Orlow

## Abstract

**Background:**

Health literacy and socioeconomic status (SES) are associated with both race/ethnicity and asthma outcomes. The extent to which health literacy and SES mediate racial/ethnic asthma disparities is less clear.

**Objective:**

To determine if health literacy and SES mediate racial/ethnic asthma disparities using advanced mediation analyses.

**Methods:**

A secondary analysis was performed using a Chicago-based longitudinal cohort study conducted from 2004 to 2007 involving 342 adults age 18 to 41 years with persistent asthma. Phone interviews were conducted every 3 months assessing asthma quality of life (AQOL; scored 1–7, with 7 being the highest) and asthma-related health care use measures. Structural equation models assessed mediation of race/ethnicity effects on AQOL and health care use through health literacy and SES. Covariates in the best-fit model included sex, year and season of interview, and cigarette smoking.

**Key Results:**

The study sample was 77.8% female, 57.3% African American/non-Hispanic, and 28.7% Hispanic. Race/ethnicity was significantly associated with AQOL and asthma-related emergency department (ED) visits, but only indirectly, through the effects of health literacy and income. Compared with White/non-Hispanics, African American/non-Hispanics and Hispanics had significantly higher odds of low health literacy and lower income. Low health literacy was associated with significantly lower AQOL scores (β = −0.24, 95% confidence interval (CI) [−0.38, −0.10]) and higher odds of an ED visit (adjusted odds ratio = 1.24, 95% CI [1.07, 1.43]). Increasing income was associated with significantly higher AQOL scores (β = 0.18, 95% CI [0.08, 0.28]) and lower odds of an ED visit (adjusted odds ratio = 0.88, 95% CI [0.80, 0.97]).

**Conclusions:**

The relationships between race/ethnicity and several asthma outcomes were mediated by health literacy and income. Interventions to improve racial/ethnic asthma disparities should target health literacy and income barriers. **[*HLRP: Health Literacy Research and Practice*. 2019;3(1):e9–e18.]**

**Plain Language Summary:**

Using advanced statistical methods, this study suggests racial/ethnic differences in several asthma outcomes are largely due to effects of health literacy and income. Interventions to improve racial/ethnic asthma disparities should target health literacy and income barriers.

Disparities in asthma outcomes are well established among racial and ethnic minorities (Gupta, Carrion-Carire, & Weiss, 2006; Haselkorn, Lee, Mink, & Weiss, 2008; Hunninghake, Weiss, & Celedon, 2006; Keller & Lowenstein, 2002; National Center for Health Statistics, 2011), although the extent to which this relationship is mediated by health literacy and socioeconomic status (SES) is less clear (Mantwill, Monestel-Umana, & Schulz, 2015). Poor health literacy, defined as “the degree to which individuals have the capacity to obtain, process, and understand basic health information and services needed to make appropriate health decisions” (Ratzan & Parker, 2000), and lower SES have both been associated with worse general health, increased health care use, and several negative asthma outcomes (Apter et al., 2013; Berkman et al., 2011; Braveman, Cubbin, Egerter, Williams, & Pamuk, 2010; Eisner, Katz, Yelin, Shiboski, & Blanc, 2001; Rosas-Salazar, Apter, Canino, & Celedon, 2012). As racial and ethnic minority populations are disproportionately affected by limited health literacy and lower SES (Morales, Lara, Kington, Valdez, & Escarce, 2002; National Center for Education Statistics, 2006), these factors may function as mediators: variables within the causal pathway that lead to worse asthma outcomes for these populations (MacKinnon, Fairchild, & Fritz, 2007).

Asthma management is complex and requires adequate self-management strategies to maintain disease control while overcoming potential social and environmental barriers. Inadequate health literacy has been associated with lower asthma knowledge, as well as worse inhaler technique and self-management (Federman et al., 2014; Mancuso & Rincon, 2006; Paasche-Orlow et al., 2005). Tailored education has the potential to overcome these barriers and may provide a target for addressing literacy-related asthma disparities (Apter, 2014; Paasche-Orlow et al., 2005). Health literacy mediates the relationship between race and several health outcomes including health status and medication adherence (Osborn et al., 2011; Osborn, Paasche-Orlow, Davis, & Wolf, 2007; Sentell & Halpin, 2006), and SES has been shown to mediate racial/ethnic health disparities among African American/non-Hispanic and Hispanic children (Schuster et al., 2012). Curtis, Wolf, Weiss, and Grammer (2012) provided preliminary evidence that health literacy and SES may mediate racial disparities in asthma-specific outcomes using simple regression-based analyses. Our study aims to add to their work by assessing mediation in the same data set using structural equation modeling (SEM), a statistical technique that permits modeling with visualization of the relationships among variables in a manner that provides an excellent framework for mediation analyses and causal inference (Gunzler, Chen, Wu, & Zhang, 2013). By using a longitudinal study design and a statistical technique better suited to infer causation, we hoped to identify mediators of racial/ethnic asthma disparities to inform interventions aimed at improving the health of minority groups.

## Methods

### Study Sample

The Chicago Initiative to Raise Asthma Health Equity (CHIRAH) was a community-based longitudinal cohort study conducted from 2004 to 2007 designed to identify factors contributing to racial and ethnic disparities in an urban area with one of the highest asthma mortality rates in the United States (Weiss et al., 2009). Detailed CHIRAH study methods have been previously published (Weiss et al., 2009). In summary, participants were recruited based on the Chicago school district (public and private) using population-proportionate and cluster sampling methods within four sampling groups of schools defined by race (greater than or less than 50% African American/non-Hispanic students) and income (greater than or less than 70% of students receiving subsidized or free lunch). Surveys were sent home with children to be filled out by the adult parent/caregiver and then returned to the teacher with a goal of identifying household members (adults age 18–41 years) with asthma. The survey completion rate was 78.9% (48,917/62,005), yielding a total of 3,676 eligible households with 5,255 possible people with asthma who then underwent telephone screening to confirm eligibility. Inclusion criteria required a history of physician-or nurse-diagnosed symptomatic asthma requiring at least 8 weeks of asthma medications during the previous year. Participants were excluded if they were not fluent in spoken English, had no telephone, or were not living in Chicago. This produced a sample size of 519 adults, of whom 353 completed the in-person baseline interview (68%). For our study, we included participants who self-identified as White/non-Hispanic, African American/non-Hispanic, or Hispanic and with health literacy measured at the baseline interview, yielding a final sample size of 342. Six follow-up phone interviews were conducted every 3 months: 71.4% of adults completed all seven interviews.

All participants gave informed consent prior to participating (Weiss et al., 2009). These analyses were approved by the Boston University Medical Center Internal Review Board.

### Measures

***Demographics/socioeconomic status.*** All demographic and SES information was collected at the baseline interview. Age, sex, race, and ethnicity were assessed, and race/ethnicity groups included Hispanic, African American/non-Hispanic, and White/non-Hispanic. SES was based on self-reported household income category (<$15,000, $15,000–$29,999, $30,000–$50,000, >$50,000), highest educational attainment, insurance status (private, Medicaid, self-pay), and employment status (full-time, part-time, not at all). For cases missing values for baseline SES variables, estimated values were obtained from the CHIRAH data set using as few assumptions as possible. All other related information on each participant was reviewed to obtain the best estimate. As this data set includes multiple interviews over time, when confronted with a missing value the first option was to impute a missing value based on other responses from that participant for that question at another interview. This was done for three variables: income, insurance, and employment status. Missing data comprised 5.8%, 0.9%, and 0.3% of these included variables, respectively. For income, there were 20 missing values for one of the survey items relating to income across the whole data set. To impute these 20 income values, ordinal logistic regression models were developed based on 333 participants with complete data.

***Health literacy.*** Health literacy was measured during the baseline interview using the Rapid Estimate of Adult Literacy in Medicine (REALM; Davis et al., 1993), which was chosen because it is brief and widely used (Berkman et al., 2011; Parker, Baker, Williams, & Nurss, 1995; Weiss et al., 2009). It has also been validated against the Test of Functional Health Literacy in Adults among a majority African American indigent population (Parker et al., 1995). For the REALM assessment, participants are asked to read aloud 66 medical terms; the REALM score is equal to the number of correctly pronounced words, with scores of 61 or above corresponding to “adequate” or “inadequate” health literacy, respectively.

***Other covariates/confounders.*** Additional covariates assessed in our model were grounded in the conceptual model exploring the causal pathways between health literacy and health outcomes (Paasche-Orlow & Wolf, 2007), limited to variables available in the data set. These included age, sex, smoking status, duration of asthma, and a year/season time variable incorporating both the year and season of each interview to account for environmental variations during the follow-up period. Smoking status was dichotomized based on current cigarette smoking status and could vary by time over the course of the study.

***Asthma outcomes.*** Asthma quality of life (AQOL) was measured using the Mini Asthma Quality of Life Questionnaire consisting of 15 items in four domains (symptoms, activity limitations, emotional function, and environmental stimuli) for the previous 2 weeks (Juniper, Guyatt, Cox, Ferrie, & King, 1999). Each item was scored on a scale of 1 (lowest) to 7 (highest), with higher scores representing better AQOL. Participants were also asked about asthma-related emergency department (ED) visits or hospitalizations in the previous 3 months. We also examined a composite use outcome, which included any asthma exacerbation requiring any type of same-day care (ED visit, hospitalization, or any same-day medical visit such as a walk-in clinic or urgent care center) in the previous 3 months. Use data obtained at the baseline interview was omitted because this assessed use during the previous year rather than the 3-month intervals inquired about at the follow-up interviews.

### Statistical Analysis

**Figure 1.**
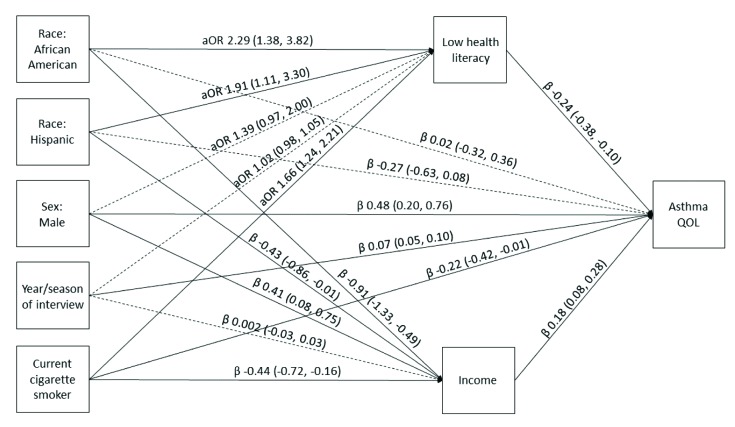
Structural equation model illustrating the mediation of race/ethnicity on asthma quality of life (QOL) through health literacy and income. Solid lines represent significant paths (*p* < .05). Regression estimates are reported as adjusted odds ratios (aOR; 95% confidence interval [CI]) for health literacy and regression beta coefficients [95% CI] for income and asthma QOL. Asthma QOL scores ranged from 1 to 7 (highest). Model fit parameters: root mean square error of approximation, 0.000 (95% CI [0.000, 0.000]); comparative fit index, 1.000; Tucker-Lewis index, 1.000; weighted root mean square residual, 0.005.

Differences in baseline characteristics by race/ethnicity were assessed by analysis of variance for continuous variables and chi-square tests for categorical variables. Structural equation models via path analysis accounting for clustering of the longitudinal data by participant identifier were used to assess mediation of race/ethnicity on AQOL and asthma health care use outcomes through health literacy level and SES variables. Time-varying data were used in a typical longitudinal manner with each participant providing multiple observations over time. Several models were evaluated for optimal fit as is done in SEM model construction by adding individual items to maximize model fit. We used standard measures of model fit (comparative fit index, the Tucker-Lewis index, the root mean square error of approximation, and weighted root mean square residual) and assessed each of the covariates. Adjusted linear regression coefficients and adjusted odds ratios (aOR) were computed from these models along with 95% confidence intervals (CI) and two-sided *p* values. The final set of independent and mediating variables included in the model and used in multivariate analyses is shown in **Figure 1**. For SES, only income improved model fit, and it was subsequently used as the representative SES variable. A linearized year-season variable provided the best model fit. SAS version 9.4 and Mplus version 7.4 were the software used for all analyses.

## Results

The total sample (*N* = 342) was 77.8% female, 57.3% African American/non-Hispanic, and 28.7% Hispanic, ranging from age 18 to 41 years with a mean (standard deviation) age of 30.9 (6.1) years (**Table 1**). Compared with White/non-Hispanics, African American/non-Hispanics and Hispanics were more likely to have low health literacy (*p* < .01), Medicaid or no insurance (*p* < .0001), and lower income (*p* < .0001). There was no difference in education level, smoking status, or duration of asthma (*p* > .05). Season of enrollment differed by race/ethnicity with African American/non-Hispanics having the greatest percentage of enrollment during the spring/summer compared with the winter/spring among Hispanics and White/non-Hispanics (*p* < .0001). The mean (standard deviation) number of completed follow-up visits (of 6 possible) was 5.4 (1.3) for the overall sample, and there was no difference between race/ethnicity groups (*p* = .56).

The unadjusted SEM regression estimates showed that African American/non-Hispanics had lower AQOL scores (β = −0.41, 95% CI [−0.75, −0.06]) and increased odds of an ED visit (odds ratio [OR] = 1.61, 95% CI [1.17, 2.21]), hospitalization (OR = 2.01, 95% CI [1.29, 3.13]), and any same-day visit of any kind (OR = 1.56, 95% CI [1.14, 2.13]) compared with White/non-Hispanics (**Table 2**). Hispanics similarly had lower AQOL scores (β = −0.44, 95% CI [−0.81, −0.07]) and increased odds of a hospitalization (OR = 1.71, 95% CI [1.08, 2.70]) compared with White/non-Hispanics, but there was no difference in ED visits or any same-day visit.

**Figure 2.**
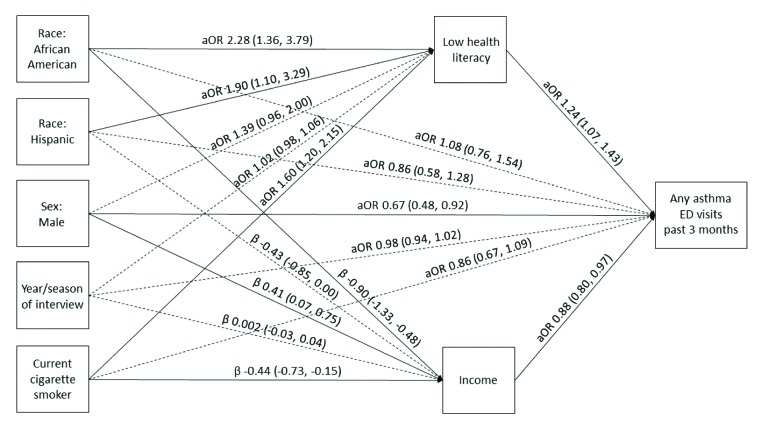
Structural equation model illustrating the mediation of race/ethnicity on any asthma-related emergency department (ED) visit in the past 3 months through health literacy and income. Solid lines represent significant paths (*p* < .05). Regression estimates are reported as adjusted odds ratios (aOR; 95% confidence interval [CI]) for health literacy and ED visits and regression beta coefficients [95% CI] for income. Model fit parameters: root mean square error of approximation, 0.000 (95% CI [0.000, 0.000]); comparative fit index, 1.000; Tucker-Lewis index, 1.000; weighted root mean square residual, 0.009.

**Figure 3.**
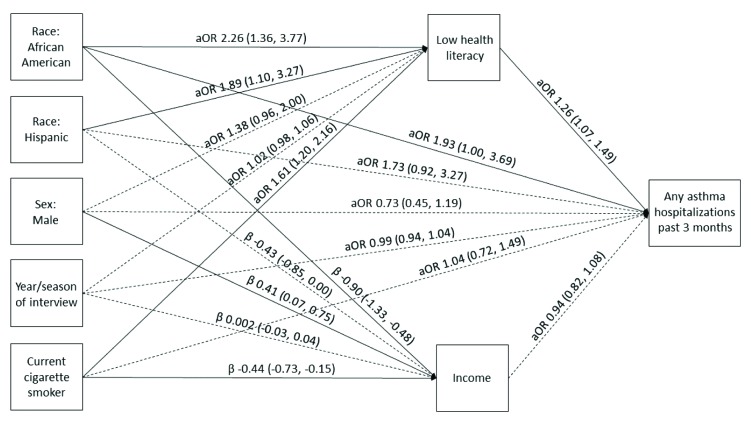
Structural equation model illustrating the mediation of race/ethnicity on any asthma-related hospitalization in the past 3 months through health literacy and income. Solid lines represent significant paths (*p* < .05). Regression estimates are reported as adjusted odds ratios (aOR; 95% confidence interval [CI]) for health literacy and hospitalizations and regression beta coefficients [95% CI] for income. Model fit parameters: root mean square error of approximation, 0.000 [0.000, 0.000]; comparative fit index, 1.000; Tucker-Lewis index, 1.000; weighted root mean square residual, 0.004.

Our models demonstrate the relationships between race/ethnicity and AQOL scores, asthma-related ED visits, and asthma hospitalizations, respectively, mediated through health literacy and income after controlling for sex, year and season of each interview, and smoking status (**Figures 1–3**). Race/ethnicity was not associated directly with AQOL score (**Figure 1**); however, low health literacy was associated with lower AQOL score (β = −0.24, 95% CI [−0.38, −0.10]; **Figure 1**), and increasing income was associated with higher AQOL score (β = 0.18, 95% CI [0.08, 0.28]; **Figure 1**). Similarly, race/ethnicity was not associated directly with ED visits (**Figure 2**), although low health literacy was associated with increased odds of an ED visit (aOR = 1.24, 95% CI [1.07, 1.43]; **Figure 2**), and increasing income was associated with decreased odds of an ED visit (aOR = 0.88, 95% CI [0.80, 0.97]; **Figure 2**). Among Hispanics, race/ethnicity was also not associated directly with hospitalizations (**Figure 3**); however, among African American/non-Hispanics, the association was borderline (aOR = 1.93, 95% CI [1.004, 3.69]; *p* = .049; **Figure 3**). Low health literacy, but not income, was associated with increased odds of hospitalization (aOR = 1.26, 95% CI [1.07, 1.49]; **Figure 3**), and a similar relationship was observed between low health literacy and any same-day asthma visit of any kind (aOR = 1.24, 95% CI [1.08, 1.43]; **Figure A**). Compared with White/non-Hispanics, African American/non-Hispanics and Hispanics had increased odds of having low health literacy (aOR = 2.29, 95% CI [1.38, 3.82]; aOR = 1.91, 95% CI [1.11, 3.30], respectively) and lower income (β = −0.91, 95% CI [−1.33, −0.49]; β = −0.43, 95% CI [−0.86, −0.01], respectively) in the AQOL model (**Figure 1**) with similar findings observed in the use models (**Figures 2–3**).

## Discussion

We have shown that racial and ethnic minorities had lower AQOL scores, increased asthma-related ED visits, asthma hospitalizations, and any type of same-day asthma visits compared with White/non-Hispanic participants using SEM and a longitudinal cohort of adults with asthma. However, these phenomena were largely mediated by health literacy and income.

Our results build on those of Curtis et al. (2012) by using a superior mediation framework with SEM applied to the same CHIRAH data set and accounting for the confounding influences of year and season, which were directly associated with AQOL and may affect asthma control due to variations in air quality and weather. This is particularly important given the nonrandom distribution of enrollment over time by race/ethnicity identified in this cohort. Controlling for age, sex, and duration of asthma, but not smoking status, Curtis et al. (2012) found that SES and health literacy explained racial differences in AQOL and ED visits but not asthma-related hospitalizations. SES in their study was comprised of income, education, insurance status, and work status, whereas income was the only SES variable in our study that improved model fit. Our study also showed a significant direct path to hospitalization for African American/non-Hispanic participants with a concurrent significant indirect path via health literacy. It has been hypothesized that unique attitudes or beliefs among racial and ethnic minorities may lead to an increased risk of hospitalization (Curtis et al., 2012), independent of SES and health literacy. Lower adherence to inhaled corticosteroids (ICS), a medication important for maintaining baseline asthma control, has been observed among African American/non-Hispanics and Hispanics compared with White/non-Hispanics (Apter et al., 2003; Federman et al., 2014; Le et al., 2008), and negative medication beliefs have been shown to mediate poor ICS adherence among racial and ethnic minorities (Le et al., 2008).

Patient, provider, and system-level factors likely interact to produce the observed asthma disparities associated with limited health literacy. Patient factors include delayed care- seeking from failing to recognize dangerous asthma symptoms, improper inhaler technique (Paasche-Orlow et al., 2005), and avoidance of routine care due to provider mistrust and a worse assessment of care quality among those with limited health literacy (Mancuso & Rincon, 2006; Paasche-Orlow & Wolf, 2007). Provider factors include failing to recognize low literacy, less time available for counseling and education, and inadequate provider teaching skills; all of which could lead to miscommunication (Paasche-Orlow & Wolf, 2007). Poor patient-provider communication is an independent predictor of poor ICS adherence in asthma management (Apter, Reisine, Affleck, Barrows, & ZuWallack, 1998). Similarly, difficulties associated with navigating the system to schedule appointments, fill medications, and comprehend insurance coverage are system-level factors that likely contribute to health care inequalities among minority groups, especially those with limited health literacy.

Our findings have implications for both research and clinical interventions. Recruitment of racial and ethnic minorities into asthma research has been limited by a failure to believe their asthma diagnosis (Kramer et al., 2016). The inability to engage vulnerable populations in research due to health literacy barriers may exacerbate disparities. Using community health workers and/or screening for social determinants may help identify socioeconomic barriers to asthma care and research (Bryant-Stephens et al., 2016). A 2012 systematic review of interventions to improve asthma outcomes among racial and ethnic minorities showed the effectiveness of education-based modalities (Press et al., 2012), likely targeting the negative impact of low health literacy. Further stratifying results of asthma intervention studies by health literacy and income may better elucidate their effectiveness among those at highest risk for poor outcomes.

## Study Limitations

Our study has several limitations. First, the generalizability of our results is limited. Our sample was restricted to relatively young, English-speaking adults in an urban area. We have no information on those who declined to participate. It is also possible that our findings would be different in other settings. The age of our data may limit present-day generalizability; although with increasing system complexity, we anticipate a larger impact of health literacy and financial barriers on health outcomes. Second, the relatively small sample size of each racial/ethnic group limited our ability to examine important aspects of diversity within each of these groups. Third, although our analyses infer causation, we are unable to reject alternative pathways related to unmeasured characteristics. Factors not available in the data set could also be in the causal chain and influence our findings. Use of controller medications, the housing environment, air pollution, and social support are factors we could not model. Finally, we used the REALM and modeled health literacy as a single construct. This is an oversimplification. Numeracy, the health literacy component related to quantitative skills, was shown to be associated with asthma-related hospitalizations and ED visits (Apter et al., 2006). Numeracy is a particularly valuable skill among adults with asthma to understand peak expiratory flow results, tapering steroids, and calculating medication doses (Rosas-Salazar et al., 2012). Additionally, health literacy should ultimately be conceptualized as a context-specific phenomenon, and therefore may be incompletely assessed using a general health literacy measure. Work is still needed to validate context-specific health literacy measures, particularly among racial and ethnic minorities (Nguyen et al., 2015). Future research exploring the role of various context-specific health literacy domains, including numeracy, in mediating racial/ethnic disparities in asthma outcomes could provide more insight into potential interventions.

## Conclusion

In conclusion, the relationships between race/ethnicity and asthma outcomes were significant and largely explained by differences in health literacy and income. There were no significant direct relationships between race/ethnicity and asthma outcomes, except hospitalizations, when these mediators were included in the models. Interventions addressing low health literacy and financial barriers among adults with asthma have the potential to improve racial/ethnic asthma disparities related to quality of life and health care use.

## Figures and Tables

**Table 1:** Baseline Characteristics of the Study Sample by Race/Ethnicity

**Characteristic**	**Total Sample (*N* = 342)**	**White/NH (*n* = 48)**	**AA/NH (*n* = 196)**	**Hispanic (*n* = 98)**	***p* Value**
Age, years (mean *SD*)^a^	30.9 (6.1)	31.9 (6.9)	30.5 (6.1)	31.3 (5.6)	.28^b^
Female, *n* (%)	266 (77.8%)	35 (72.9%)	159 (81.1%)	72 (73.5%)	.23^c^
Education, *n* (%)					.46^c^
Some high school	56 (16.4%)	5 (10.4%)	33 (16.8%)	18 (18.4%)	
High school graduate/GED	112 (32.8%)	18 (37.5%)	68 (34.7%)	26 (26.5%)	
Some college	174 (50.9%)	25 (52.1%)	95 (48.5%)	54 (55.1%)	
Insurance status, *n*^d^ (%)					<.0001^c*^
Private	155 (45.4%	36 (76.6%)	67 (34.2%)	52 (53.1%)	
Medicaid	141 (41.4%)	9 (19.2%)	100 (51.0%)	32 (32.7%)	
Self-pay	45 (13.2%)	2 (4.3%)	29 (14.8%)	14 (14.3%)	
Income, *n* (%)					<.0001^c*^
<$15,000	96 (28.1%)	7 (14.6%)	71 (36.2%)	18 (18.4%)	
$15,000–$29,999	86 (25.2%)	5 (10.4%)	54 (27.5%)	27 (27.5%)	
$30,000–$50,000	64 (18.7%)	8 (16.7%)	36 (18.4%)	20 (20.4%)	
>$50,000	96 (28.1%)	28 (58.3%)	35 (17.9%)	33 (33.7%)	
Employment, *n*^d^ (%)					.02^c*^
Full-time	143 (42.4%)	21 (43.8%)	74 (38.3%)	48 (50%)	
Part-time	63 (18.7%)	10 (20.8%)	30 (15.5%)	23 (24%)	
None	131 (38.9%)	17 (35.4%)	89 (46.1%)	25 (26%)	
Current smoking status, *n*^d^ (%)					.87^c^
Nonsmoker	237 (69.7%)	35 (72.9%)	135 (69.2%)	68 (69.1%)	
Smoker	103 (30.3%)	13 (27.1%)	61(30.8%)	30 (30.9%)	
Asthma duration, years (mean *SD*)	18.0 (10.4)	16.5 (12.1)	18.2 (10.3)	18.4 (9.7)	.53^b^
Year of enrollment, *n* (%)					<.0001^c*^
2004	162 (47.4%)	12 (25%)	128 (65.3%)	22 (22.5%)	
2005	180 (52.6%)	36 (75%)	68 (34.7%)	76 (77.5%)	
Season of enrollment, *n* (%)					<.0001^c*^
Winter	75 (21.9%)	21 (43.8%)	20 (10.2%)	34 (34.7%)	
Spring	140 (40.9%)	20 (41.7%)	80 (40.8%)	40 (40.8%)	
Summer	84 (24.6%)	6 (12.5%)	66 (33.7%)	12 (12.2%)	
Fall	43 (12.6%)	1 (2.1%)	30 (15.3%)	12 (12.2%)	
Visits completed (mean *SD*)^e^	5.4 (1.3)	5.2 (1.5)	5.4 (1.2)	5.4 (1.3)	0.56^c^
Low health literacy, *n* (%)	110 (32.2%)	6 (12.5%)	74 (37.8%)	30 (30.6%)	<.01^c*^

Note. AA = African American; GED = general education development; NH = non-Hispanic.

a Study sample age ranged from 18 to 41 years based on sample selection.

b Analysis of variance test.

c Chi-square test.

d Missing data accounted for *n* = 1, *n* = 5, and *n* = 2 in the total sample for insurance, employment, and smoking status, respectively.

e Maximum number of follow-up visits was six.

* *p* < .05.

**Table 2:** Unadjusted Asthma Outcomes by Race/Ethnicity

**Asthma Outcome**	**White/NH (*n* = 48)**	**AA/NH (*n* = 196)**	**Hispanic (*n* = 98)**
Mean AQOL score (beta [95% CI])^a^	1.00 (ref)	−0.41 (−0.75, −0.06)^*^	−0.44 (−0.81, −0.07)^*^
Any asthma ED visit in past 3 months (OR [95% CI])	1.00 (ref)	1.61 (1.17, 2.21)^**^	1.16 (0.82, 1.64)
Any asthma hospitalization in past 3 months (OR [95% CI])	1.00 (ref)	2.01 (1.29, 3.13)^**^	1.71 (1.08, 2.70)^*^
Any same-day asthma visit of any kind in past 3 months (OR [95% CI])^b^	1.00 (ref)	1.56 (1.14, 2.13)^**^	1.27 (0.91, 1.78)

Note. AA = African American; AQOL = asthma quality of life; ED = emergency department; NH = non-Hispanic; OR = odds ratio; ref = reference group.

a AQOL score ranges from 1 to 7. Higher score represents higher AQOL.

b Includes asthma-related ED visits, hospitalizations, walk-in visits, or urgent care visits.

* *p* < .05.

** *p* < .01.

**Figure A.**
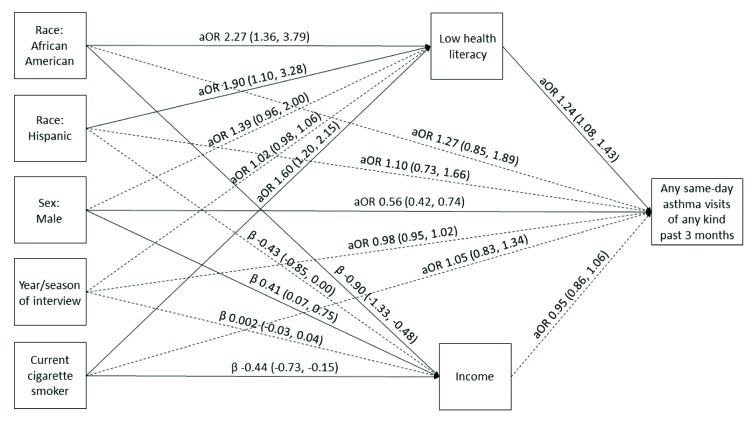
Structural equation model illustrating the mediation of race/ethnicity on any same-day asthma visit through health literacy and income. Same-day asthma visit was defined as an emergency department visit, hospitalization, or other same-day medical visit such as a walk-in clinic or urgent care center. Solid lines represent significant paths (*p* < .05). Regression estimates are reported as adjusted odds ratios (aOR; 95% confidence interval [CI]) for health literacy and same-day asthma visits and regression beta coefficients [95% CI] for income.
